# The Past and Future of Tuberculosis Research

**DOI:** 10.1371/journal.ppat.1000600

**Published:** 2009-10-26

**Authors:** Iñaki Comas, Sebastien Gagneux

**Affiliations:** Division of Mycobacterial Research, MRC National Institute for Medical Research, London, United Kingdom; The Scripps Research Institute, United States of America

## Abstract

Renewed efforts in tuberculosis (TB) research have led to important new insights into the biology and epidemiology of this devastating disease. Yet, in the face of the modern epidemics of HIV/AIDS, diabetes, and multidrug resistance—all of which contribute to susceptibility to TB—global control of the disease will remain a formidable challenge for years to come. New high-throughput genomics technologies are already contributing to studies of TB's epidemiology, comparative genomics, evolution, and host–pathogen interaction. We argue here, however, that new multidisciplinary approaches—especially the integration of epidemiology with systems biology in what we call “systems epidemiology”—will be required to eliminate TB.

## Introduction

**Figure 1:**
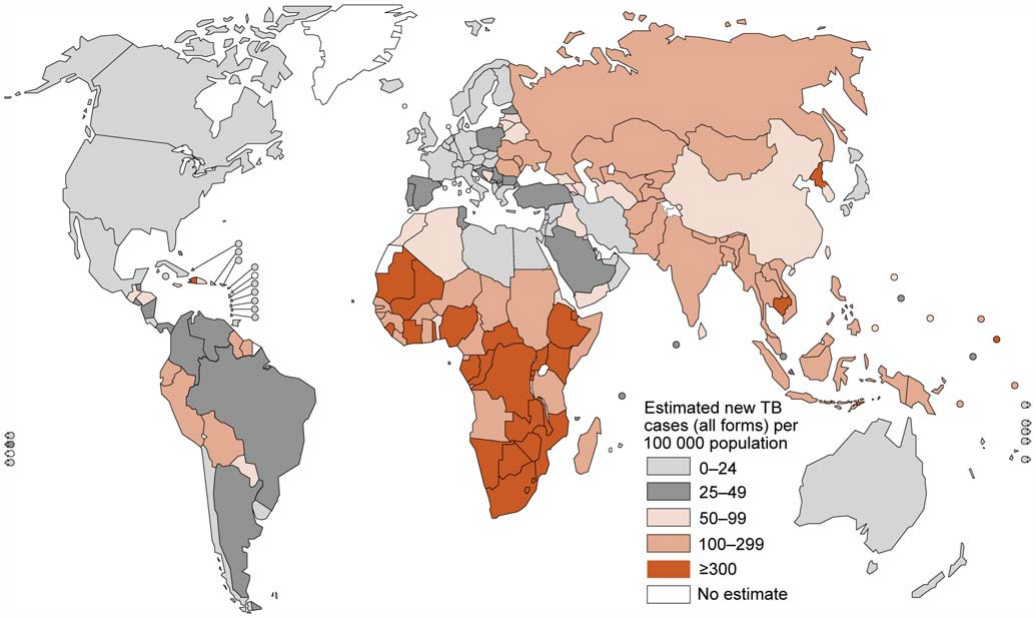
The global incidence of TB. The number of new TB cases per 100,000 population for the year 2007 according to WHO estimates (adapted from [1]).

Tuberculosis (TB) remains an important public health problem [1]. With close to 10 million new cases per year, and a pool of two billion latently infected individuals, control efforts are struggling in many parts of the world (Figure 1). Nevertheless, the renewed interest in research and improved funding for TB give reasons for optimism. Recently, the Stop TB Partnership, a network of concerned governments, organizations, and donors lead by the WHO (http://www.stoptb.org/stop_tb_initiative/), outlined a global plan to halve TB prevalence and mortality by 2015 and eliminate the disease as a public health problem by 2050 [2].Attaining these goals will depend on both strong government commitment and increased interdisciplinary research and development. As existing diagnostics, drugs, and vaccines will be insufficient to achieve these objectives, a substantial effort in both basic science and epidemiology will be necessary to develop better tools and strategies to control TB [3]. Here we review the recent history of TB research and some of the latest insights into the evolutionary history of the disease. We then discuss ways in which we could benefit from a more comprehensive systems approach to control TB in the future.

## Recent History of the Field

TB is caused by several species of gram-positive bacteria known as tubercle bacilli or *Mycobacterium tuberculosis* complex (MTBC). MTBC includes obligate human pathogens such as *Mycobacterium tuberculosis* and *Mycobacterium africanum*, as well as organisms adapted to various other species of mammal. In the developed world, TB incidence declined steadily during the second half of the 20th century and so funds available for research and control of TB decreased substantially during that time [4]. When TB started to reemerge in the early 1990s, fuelled by the growing pandemic of HIV/AIDS (Box 1), scientists and public health officials were caught off-guard; billions of dollars of emergency funds were necessary to control TB outbreaks [5]. Moreover, long-term neglect of basic TB research and product development meant that global TB control relied on a 100-year-old diagnostic method (i.e. sputum smear microscopy) of poor sensitivity, an 80-year-old and largely ineffective vaccine (Bacille Calmette-Guérin [BCG]), and just a few drugs that were decades old (streptomycin, rifampicin, isoniazid, ethambutol, pyrozinamide) [3]. Tragically, these are the tools still in use today in most parts of the world where TB remains one of the most important public health problems (Figure 1).

**Box 1. The Influence of Modern Epidemics on TB Incidence**

HIV/AIDS and diabetes are important comorbidities that dramatically increase the susceptibility to TB. The synergy between TB and HIV/AIDS is a particular problem in sub-Saharan Africa, while the impact of diabetes on TB is increasing in many rapidly growing world economies; it may already be a more important risk factor for TB than HIV/AIDS in places like India and Mexico. The emergence of multidrug-resistant strains represents an additional threat to global TB control. The strong association between HIV/AIDS and drug-resistant TB has been well established, but whether similar interactions exist between drug-resistant TB and diabetes needs to be explored further.

In addition to the lack of appropriate tools to control TB globally, much about the disease was unknown in the early 1990s and many dogmas were guiding the field at the time. These included the view that differences in the clinical manifestation of TB were primarily driven by host variables and the environment as opposed to bacterial factors, a notion reinforced by early DNA sequencing studies that reported very limited genetic diversity in MTBC compared with other bacterial pathogens [6]. According to other dogmas, TB was mainly a consequence of reactivation of latent infections rather than ongoing disease transmission, and that mixed infections and exogenous reinfections with different strains were very unlikely.

The development of molecular techniques to differentiate between strains of MTBC made it possible to readdress some of these points. One of these methods, a DNA fingerprinting protocol based on the *Mycobacterium* insertion sequence IS6110, quickly evolved into the first international gold standard for genotyping of MTBC [7]. It also became a key component of pragmatic public health efforts, such as detecting disease outbreaks and ongoing TB transmission [8], and allowed differentiation between patients who relapsed due to treatment failure and those reinfected with a different strain [9]. This latter finding demonstrated for the first time that previous exposure to MTBC does not protect against subsequent exogenous reinfection and TB disease, which is a phenomenon with implications for vaccine design. Many other new insights were gained through these molecular epidemiological studies [10], which, for the most part, were performed in wealthy countries; corresponding data from most high-burden areas remained limited because of poor infrastructure and lack of funding.

Routine genotyping of MTBC for public health purposes also revived discussions about the role of pathogen variation in outcome of infection and disease. Some strains of MTBC appeared over-represented in particular patient populations, which suggested that strain diversity may have epidemiological implications. The completion of the first whole genome sequence of *M. tuberculosis* in 1998 [11] and the development of DNA microarrays offered a new opportunity to address this question by interrogating the entire genome of multiple clinical strains of MTBC. These comparative genomics studies revealed that genomic deletions, also known as large sequence polymorphisms (LSPs), are an important source of genome plasticity in MTBC [12]. Furthermore, statistical analyses of patient data suggested possible associations between strain genomic content and disease severity in humans [13]. Clinical phenotypes in TB are difficult to standardize, however, and whether MTBC genotype plays a meaningful role in TB severity remains controversial [14].

**Figure 2:**
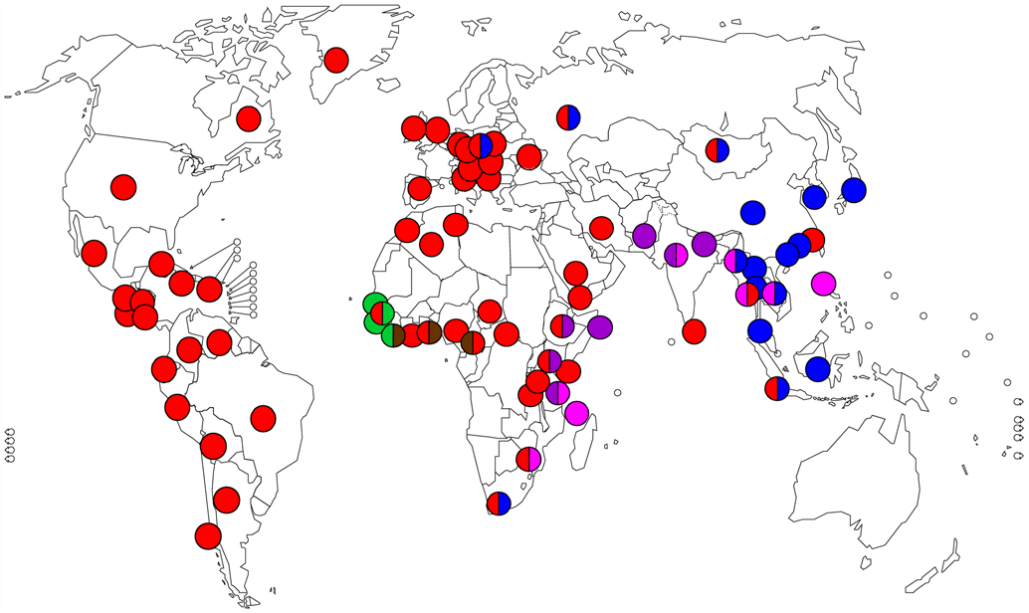
Global distribution of the six main lineages of human MTBC. Each dot represents the most frequent lineage(s) circulating in a country. Colours correspond to the lineages defined in Figure 3 (adapted from [20]).

**Figure 3:**
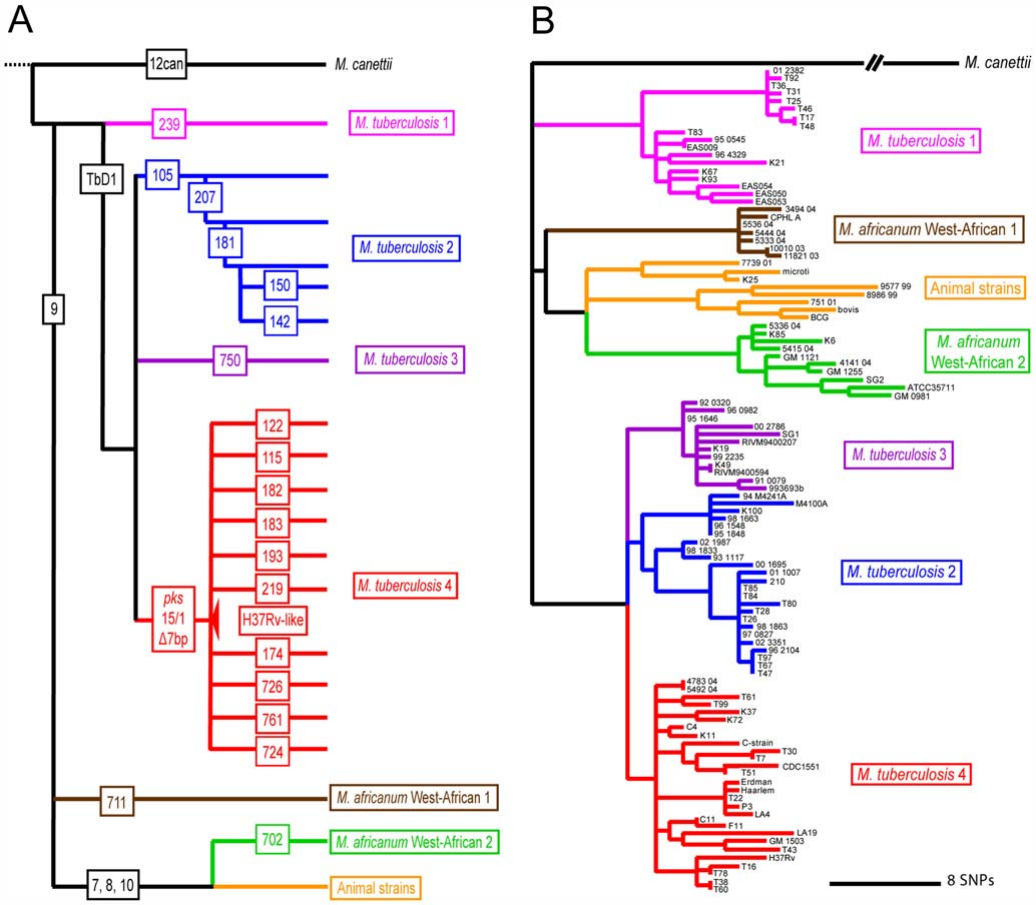
The global phylogeny of *Mycobacterium tuberculosis* complex (MTBC). The phylogenic relationships between various human- and animal-adapted strains and species are largely consistent when defined by using either (A) large sequence polymorphisms (LSPs) or (B) single nucleotide polymorphisms (SNPs) identified by sequencing 89 genes in 108 MTBC strains. Numbers inside the squares in (A) refer to specific lineage-defining LSPs. Colors indicate congruent lineages (adapted from [20] and [29]).

Comparative genomics of MTBC also yielded interesting insights into the evolution and geographic distribution of the organism. Because MTBC has essentially no detectible horizontal gene transfer [15],[16], LSPs can be used as phylogenetic markers to trace the evolutionary relationships of different strain families. Following such an approach, studies have shown that humans did not, as previously believed, acquire MTBC from animals during the initiation of animal domestication, rather the human- and animal-adapted members of MTBC share a common ancestor, which might have infected humans even before the Neolithic transition [17],[18]. LSPs also allowed researchers to define several discrete strain lineages within the human-adapted members of MTBC, which are associated with different human populations and geographical regions (Figures 2 and 3) [15],[19],[20]. Because of the lack of horizontal gene exchange in MTBC, phylogenetic trees derived using various molecular markers define the same phylogenetic groupings [21], and several studies based on single nucleotide polymorphisms (SNPs) and other molecular makers have gathered additional support for the highly phylogeographical population structure of MTBC [22]–[25].

## Ancient History of the Pathogen

Although LSPs have proven very useful for defining different lineages within MTBC, these markers do not reflect actual genetic distances, and the mode of molecular evolution in MTBC cannot be easily inferred from them [21]. By contrast, DNA sequence-based methods can provide important clues about the evolutionary forces shaping bacterial populations. Multilocus sequence typing (MLST), in which fragments of seven structural genes are sequenced for each strain [26], has been used very successfully to define the genetic population structure of many bacterial species [27]. Because of the low degree of sequence polymorphisms in MTBC, however, standard MLST is uninformative [28]. A recent study of MTBC extended the traditional MLST scheme by sequencing 89 complete genes in 108 strains, covering 1.5% of the genome of each strain [29]. Phylogenetic analysis of this extended multilocus sequence dataset resulted in a tree that was highly congruent with that generated previously using LSPs (Figure 3). The new sequence-based data also revealed that the MTBC strains that are adapted to various animal species represent just a subset of the global genetic diversity of MTBC that affects different human populations [29]. Furthermore, by comparing the geographical distribution of various human MTBC strains with their position on the phylogenetic tree, it became evident that MTBC most likely originated in Africa and that human MTBC originally spread out of Africa together with ancient human migrations along land routes. This view is further supported by the fact that the so-called “smooth tubercle bacilli,” which are the closest relatives of the human MTBC, are highly restricted to East Africa [30]. The multilocus sequence data reported by Hershberg et al. [29] further suggested a scenario in which the three “modern” lineages of MTBC (purple, blue, and red in Figure 3) seeded Eurasia, which experienced dramatic human population expansion in more recent times. These three lineages then spread globally out of Europe, India, and China, respectively, accompanying waves of colonization, trade and conquest. In contrast to the ancient human migrations, however, this more recent dispersal of human MTBC occurred primarily along water routes [29].

The availability of comprehensive DNA sequence data has also allowed researchers to address questions about the molecular evolution of MTBC. In-depth population genetic analyses by Hershberg et al. highlight the fact that purifying selection against slightly deleterious mutations in this organism is strongly reduced compared to other bacteria [29]. As a consequence, nonsynonymous SNPs tend to accumulate in MTBC, leading to a high ratio of nonsynonymous to synonymous mutations (also known as dN/dS). The authors hypothesized that the high dN/dS in MTBC compared to most other bacteria might indicate increased random genetic drift associated with serial population bottlenecks during past human migrations and patient-to-patient transmission. If confirmed, this would indicate that “chance,” not just natural selection, has been driving the evolution of MTBC. Although these kinds of fundamental evolutionary questions are often underappreciated by clinicians and biomedical researchers, studying the evolution of a pathogen ultimately allows for better epidemiological predictions by contributing to our understanding of basic biology, particularly with respect to antibiotic resistance.

## A Vision for the Future

Thanks to recent increases in research funding for TB [4], substantial progress has been made in our understanding of the basic biology and epidemiology of the disease. Unfortunately, this increased knowledge has not yet had any noticeable impact on the current global trends of TB (Figure 1). While TB incidence appears to have stabilized in many countries, the total number of cases is still increasing as a function of global human population growth [1]. Of particular concern are the ongoing epidemics of multidrug-resistant TB [31], as well as the synergies between TB and the ongoing epidemics of HIV/AIDS and other comorbidities such as diabetes (Box 1).

**Figure 4:**
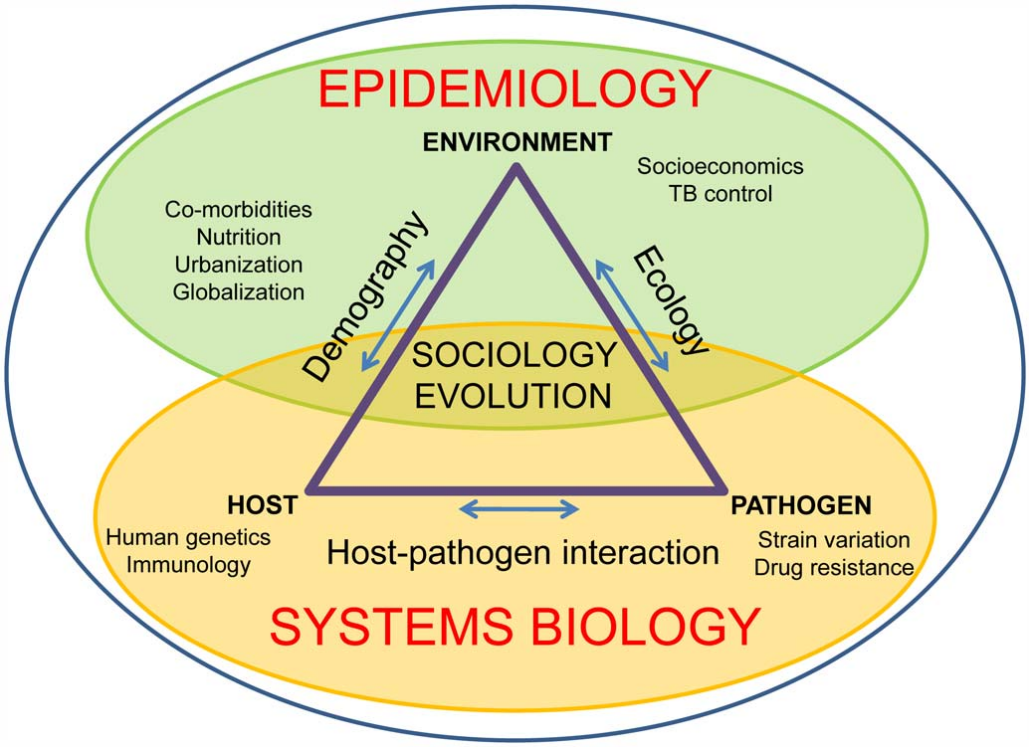
A systems epidemiology approach to TB research. The spread of TB is influenced by social and biological factors. On the one hand, the new discipline of systems biology integrates approaches that address the host, the pathogen, and interactions between the two. On the other hand, epidemiology addresses the burden of the disease and the social, economic, and ecological causes of its frequency and distribution. There is little crosstalk between these two disciplines at the moment. “Systems epidemiology” is an attempt to take into account the interactions between these various fields of research.

As our understanding of TB improves, we would like to be able to make better predictions about the future trajectory of the disease and to develop new tools to control the disease better and ultimately reverse global trends. For this to be feasible, TB epidemiology needs to evolve into a more predictive, interdisciplinary endeavour; a discipline we might refer to as “systems epidemiology” (Figure 4). Systems biology is already a rapidly emerging field, in which cycles of mathematical modelling and experiments using various large-scale “-omics” datasets are integrated in an iterative manner [32]. Novel biological processes are being discovered through these systems approaches, which might not have been possible using more traditional methods [33]–[35].

Last year, Young et al. argued that systems biology approaches will be necessary to elucidate some of the key aspects of host–pathogen interactions in TB [36] and to develop new drugs, vaccines, and biomarkers to evaluate new interventions [3]. For example, according to another dogma in the TB field, latent TB infections are caused by physiologically dormant bacilli and can thus be differentiated from active disease where MTBC is actively growing and dividing [37]. In reality, however, the phenomenon of TB latency most likely reflects a whole spectrum of responses to TB infection, involving phenotypically distinct bacterial subpopulations and spanning various degrees of bacterial burden and associated host immune responses [38]. We agree with Young et al. [36] that TB latency and similar biological complexities will only be adequately addressed using systems approaches, and we argue further that to comprehend the current TB epidemic as a whole, and to better predict its future trajectory, a complementary systems epidemiology approach will be necessary (Figure 4).

Mathematical models are already being used extensively to study the epidemiology of TB and to guide control policies [39]. Recent applications have shown that socioeconomic factors are key drivers of today's TB epidemic [40]. In addition, much theoretical emphasis has been placed on trying to define the impact that drug resistance will have on the global TB epidemic [41]. Some of this theoretical work has become more complex by incorporating new biological insights obtained empirically and through targeted experimental studies. Early theoretical studies on the spread of drug-resistant MTBC were based on the assumption that all drug-resistant bacteria had an inherent fitness disadvantage compared to drug-susceptible strains [42]; however, as is becoming clear from experimental and molecular epidemiological investigation, substantial heterogeneity exists with respect to the reproductive success of drug-resistant strains [43]–[46]. Newer mathematical models account for some of this heterogeneity [47]–[49].

One could imagine an expansion of such mathematical approaches—much as systems biology operates—in which epidemiological modelling is combined with more comprehensive biological data related to the host, the pathogen, and their interactions (Figure 4). Of course, environmental and sociological data would also need to be considered [40]. As mathematical models become more finely tuned, they could in turn inform future experimental work to test some of the specific predictions. The genomics revolution now offers the opportunity to study host–pathogen interactions at an unprecedented depth. To be able to make sense out of the current and upcoming deluge of -omics data, however, scientists will have to rely on a mathematically and statistically robust analytical framework. Ideally, some of these theoretical approaches will be able to accommodate increasingly diverse sets of data in order to capture the various biological, environmental, and social aspects of TB.

Among the newly emerging technologies, we believe that next-generation DNA sequencing will play an important role in improving our understanding of TB [50]. Whole-genome sequencing could potentially become the new gold standard for strain typing in routine molecular epidemiology [51]. For host genetics and TB susceptibility, too, de novo DNA sequencing based approaches could have advantages over traditional SNP typing [52]. For example, many of the human populations carrying the largest proportion of the global TB burden have not been sufficiently characterised genetically (Figure 1) [53],[54], and screening for currently limited human SNP collections might have little relevance for these populations [55]. Furthermore, comprehensive DNA sequencing of TB patients and controls in various human populations could help unveil rare but biologically relevant mutations [56]. Another approach increasingly being used to study both the host and the pathogen is sequence-based transcriptomics, in which gene expression is measured by whole genome sequencing of RNA transcripts; a method referred to as RNA-seq [57]. One of the advantages of this approach over existing microarray-based methods is that changes in the expression of noncoding RNAs and other novel transcripts can be easily detected. RNA-seq is particularly useful for genome-wide studies of small regulatory RNAs, as such studies are more difficult to perform using standard DNA microarrays. Recent studies, for example, have reported a role for small regulatory RNAs in *M. tuberculosis* [58], and there is little doubt more regulatory RNAs will soon be identified by RNA-seq [57].

## Challenges for the Future

Advances in TB research are hampered by the fact that MTBC is a Biosafety Level 3 pathogen with a long generation time, making it slow and complex to culture. Moreover, TB is a chronic disease that can develop over many years, and is characterised by extended periods of latency during which MTBC cannot be isolated from infected individuals. All of these factors complicate and prolong the development of new interventions and their assessment in clinical trials. As we have already mentioned, the field has been marked by a number of dogmas that, in some cases, might have contributed to the slow progress in TB research. New insights are now questioning some of these views, but at the same time, new opinions could well evolve into new dogmas. For example, we and others have spent much of our scientific careers seeking convincing evidence for the role of MTBC strain diversity in human disease. Although some pieces of evidence have recently started to emerge [59]–[61], the subject needs more work. One of the problems has been that the macrophage and mouse infection models used in these studies relied on poorly characterised strains, and finding relevant links to human disease has been all but impossible [14],[21].

In TB control, too, potential new dogmas might emerge to limit future progress. A strong T cell–derived interferon gamma (INFγ) response appears to be crucial for the immunological control of TB, and many MTBC antigens have been identified based on their capacity to elicit INFγ responses in TB patients or their infected contacts [62]. Some of these antigens are being developed into new TB diagnostics and vaccines, but the potential impact of MTBC diversity on immune responses is not generally being considered [21]. A recent study in The Gambia showed that INFγ responses to one of the key MTBC antigens differed in an MTBC lineage–specific manner [63]. Developing a universally effective vaccine might be the only way to eliminate TB in the future [3]. This is particularly true given the large reservoir of latently infected individuals in the world, which would be impossible to eliminate through prophylactic drug treatment. Considering that natural TB infection does not protect against exogenous reinfection and disease, however, mimicking natural infection using attenuated strains or a cocktail of traditional INFγ-inducing antigens might not necessarily be the most promising vaccine strategy. Indeed, the largely unsuccessful implementation of BCG vaccination might serve as a warning [64].
